# Big Cats in Our Backyards: Persistence of Large Carnivores in a Human Dominated Landscape in India

**DOI:** 10.1371/journal.pone.0057872

**Published:** 2013-03-06

**Authors:** Vidya Athreya, Morten Odden, John D. C. Linnell, Jagdish Krishnaswamy, Ullas Karanth

**Affiliations:** 1 Wildlife Conservation Society-India, Centre for Wildlife Studies, Bangalore, Karnataka, India; 2 Faculty of Applied Ecology and Agricultural Sciences, Hedmark University College, Campus Evenstad, Elverum, Norway; 3 Norwegian Institute for Nature Research, Trondheim, Norway; 4 Ashoka Trust for Research in Ecology and the Environment, Bangalore. Karnataka, India; Australian Wildlife Conservancy, Australia

## Abstract

Protected areas are extremely important for the long term viability of biodiversity in a densely populated country like India where land is a scarce resource. However, protected areas cover only 5% of the land area in India and in the case of large carnivores that range widely, human use landscapes will function as important habitats required for gene flow to occur between protected areas. In this study, we used photographic capture recapture analysis to assess the density of large carnivores in a human-dominated agricultural landscape with density >300 people/km^2^ in western Maharashtra, India. We found evidence of a wide suite of wild carnivores inhabiting a cropland landscape devoid of wilderness and wild herbivore prey. Furthermore, the large carnivores; leopard *(Panthera pardus)* and striped hyaena (*Hyaena hyaena*) occurred at relatively high density of 4.8±1.2 (sd) adults/100 km^2^ and 5.03±1.3 (sd) adults/100 km^2^ respectively. This situation has never been reported before where 10 large carnivores/100 km^2^ are sharing space with dense human populations in a completely modified landscape. Human attacks by leopards were rare despite a potentially volatile situation considering that the leopard has been involved in serious conflict, including human deaths in adjoining areas. The results of our work push the frontiers of our understanding of the adaptability of both, humans and wildlife to each other’s presence. The results also highlight the urgent need to shift from a PA centric to a landscape level conservation approach, where issues are more complex, and the potential for conflict is also very high. It also highlights the need for a serious rethink of conservation policy, law and practice where the current management focus is restricted to wildlife inside Protected Areas.

## Introduction

Charismatic predatory species have long held a central place in global conservation strategies, both in terms of attracting the public’s attention and serving as a focus for research and conservation effort [1]. Accordingly, they are frequently cast in the roles of flagships and umbrellas [2]–[4]. This is based on symbolic (their flagship role) and functional (their umbrella role as mediators of top-down cascade effects) perception of their role in ecosystem processes and their presumed dependence on wild nature and wilderness. A consequence of this is that strategic planning for their conservation is often based around protected areas that by design largely exclude or minimize inclusion of populated areas with human settlements and agricultural land-use. The protected area focus for large predator conservation has been a powerful argument for justifying the setting aside of wilderness areas, especially in tropical developing countries [5] where there has been a long standing skepticism about the ability of wildlife to persist in unprotected landscapes with moderate to high human densities [6]. India is no exception to this pattern, and most of its conservation focus, in terms of conservation actions, research, and legislation, is focused on protected areas which aim to minimize human settlements and agro-pastoral land-use. In some cases, such as tiger (*Panthera tigris*) conservation, this priority given to protected areas may well be justified [7], [8].

Some ecological studies, albeit in areas with sparse human density, have documented that large predators in general [9]–[11] benefit from areas with high densities of their natural, wild prey. Although there is also some evidence of carnivores that adapt to a wide variety of habitats even with a long history of human impacts and influences [12]. What is important for a pragmatic approach to the conservation of these wide ranging carnivores is to identify the limits of their tolerance, as much as the nature of their preferences, and these can be assessed only when the large carnivores occur outside protected areas, where their interface with humans is high. This knowledge is crucial when planning for long term conservation objectives which will need to integrate these species into the wider landscapes matrices where protected areas [8], [13] can be connected via human use landscapes.

During the last twenty years, there has been an increasing awareness of the ability of some large predators like wolves (*Canis lupus*) and pumas (*Puma concolor*) to live in very human-dominated, even sub-urban, environments in the developed world [14], [15]. Accordingly there has been a great deal of research aimed at these situations and appropriate management responses have been developed [15].

Most examples of large carnivores in urban landscapes are from countries with a low human population density although in the case of mountain lions in Southern California, it has been seen that part of their home ranges overlap with densely populated urban landscapes [15]. Recent results from Africa show comparable densities for lions, cheetahs and jackals inside and outside the protected areas, although the human population density there was low [12]. Very little is known about the ecology of ‘urban’ carnivores in densely populated countries where the potential for conflict can be very high. For instance, India has a high diversity of large carnivores, many of which share spaces with one of the highest human and livestock populations in the world [16]–[18]. The most common is the leopard (*Panthera pardus)*, which is frequently reported from many human dominated landscapes across India where it is involved in a wide range of conflict situations, often with fatal outcomes for humans.

The main management response, in the absence of robust information on large carnivore ecology in human dominated areas has been their translocation to nearby protected areas [19] based on a belief that these leopards are “stray” individuals that have dispersed from protected forest areas and need to be “helped” back to the forests. Recently, this has been documented to worsen the situation, leading to increased attacks on people near the sites of release [18]. Clearly there is a need to document the status of leopards living in these human-dominated landscapes, as well as to understand the community structure of wild carnivores in highly modified ecosystems that are also home to high density of humans. In this paper, we present evidence of the presence of an entire community of wild carnivores that share space with very high densities of humans. Although the work focused on estimating the density of the leopard populations, we also provide a density estimate of the striped hyaena (*Hyaena hyaena*) and an overview of the occurrence of other carnivore species. Using the results of this work, we discuss the potential importance of broadening the current protected area conservation focus to include a consideration of the value of human-dominated landscapes [20].

## Methods

### Study Area

**Figure 1:**
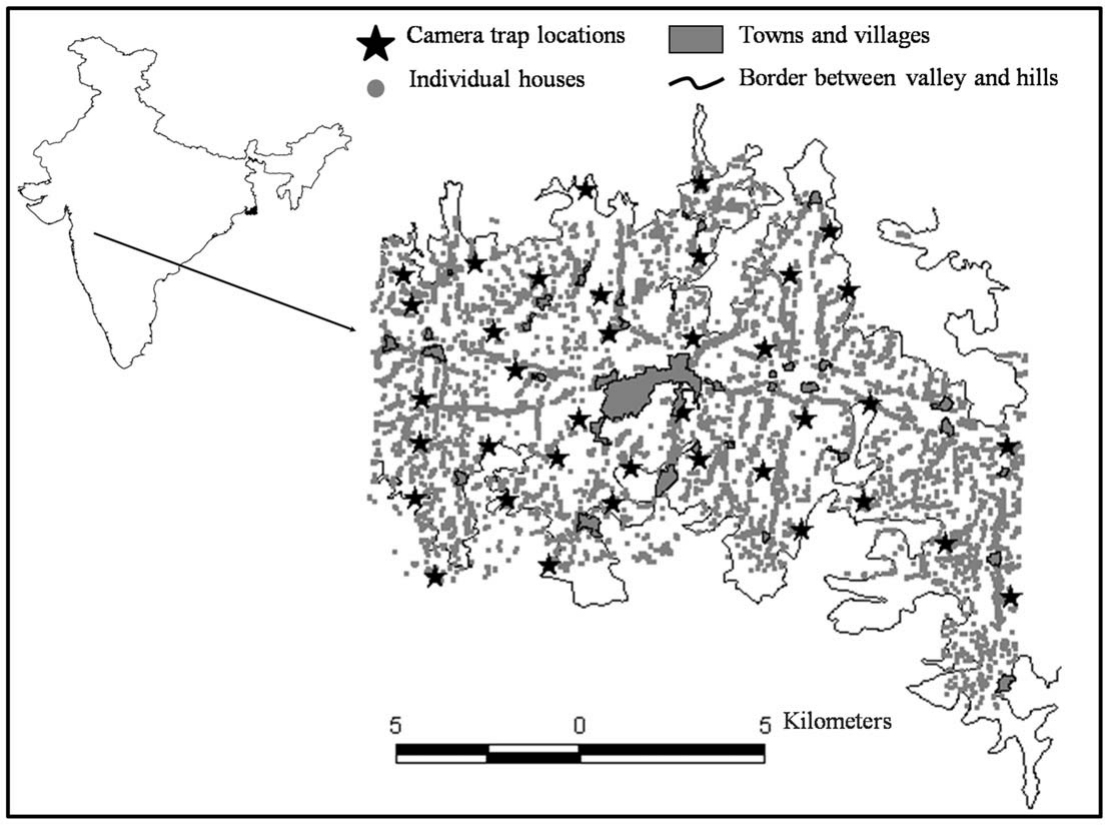
Map of the study area which consisted of an irrigated valley around the town of Akole in the Ahmednagar District, Maharashtra, India.

**Figure 2:**
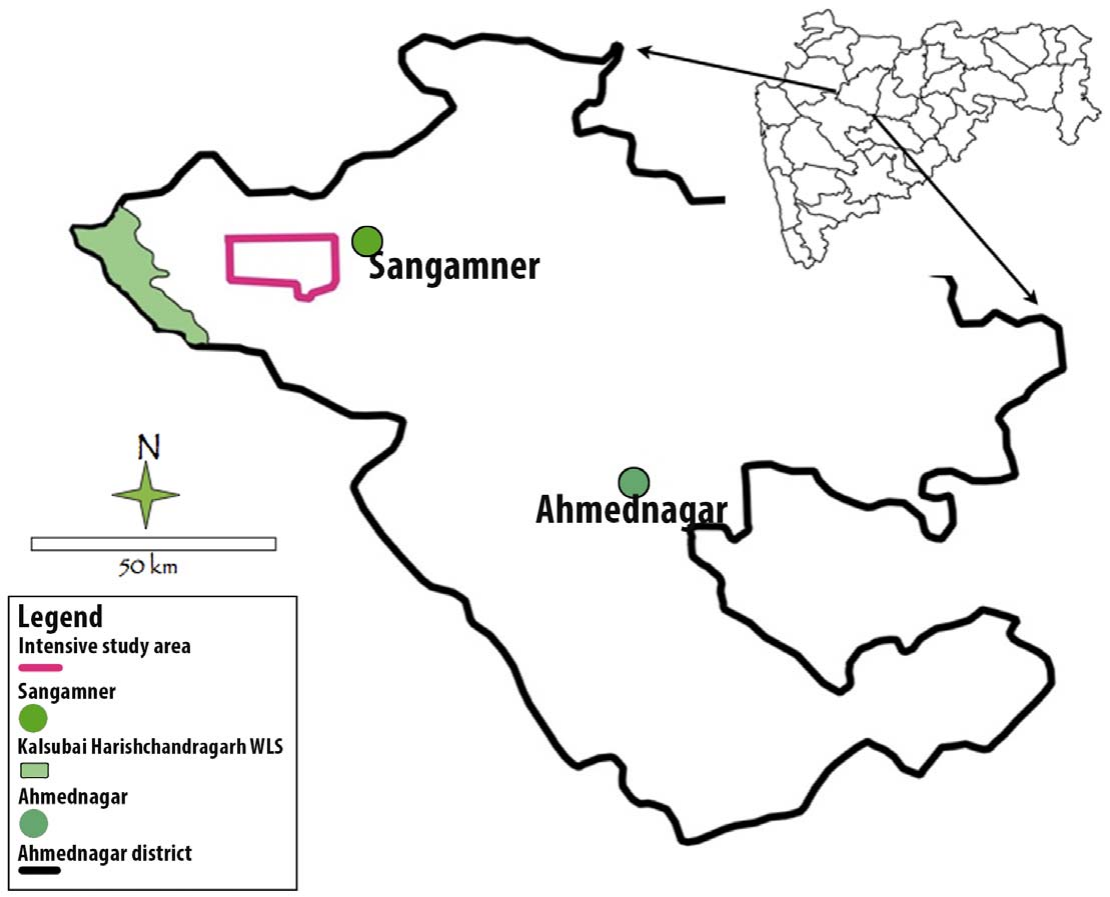
Map of the Ahmednagar district with the study area polygon and the nearest protected area of Kalsubai Harishchandragarh Wildlife Sanctuary.

The study was conducted in a densely populated, irrigated valley in Akole Tahsil located in the Ahmednagar district (19.576959 N 73.937123 E to 19.460715 N 74.089954 E) of western Maharashtra, India. An Indian district is administratively comparable to a ‘county’ and the Tahsil is a sub-unit of it. Approximately 80% of the human population is rural with farming of sugar cane, millets, and vegetables being the major source of livelihood. Rainfall varies from 1000 to 2000 mm per year. Akole Tahasil contains 191 villages (as per the 2001 census; http://ahmednagar.gov.in/html_docs/GEO-Main.htm accessed 26th June 2012) with a human population density of 177 km^−2^. The intensive study area covered a 179 km^2^ area in the irrigated valley around Akole town. Digitized maps of all the households in the study area, combined with household interviews were used to determine the average number of people per household (Athreya et al. unpublished) which was 357 humans/km^2^ in the intensively cultivated area. Only 15% (1515 km^2^) of Akole Tahasil is protected (legally designated as government-owned Reserved Forest; [21]), and mainly occurs in its western part or in small, dispersed patches unsuitable for cultivation (see Figure 1). The only wildlife protected area is the Kalsubai Harishchandragarh Wildlife Sanctuary (299 km^2^), which is located on the western boundary of Akole Tahasil, 18 km beyond the edge of our intensive study area (Figure 2).

### Ethics Statement

Relevant permissions to carry out the ecological research were obtained from the Office of the Chief Wildlife Warden, Maharashtra Forest Department.

### Camera Trap Surveys

Forty camera trap locations were selected following study design approaches prescribed for large felids [22] at sites that had intensive signs of their usage. Most trap sites were on human trails. The duration of the camera trap survey was fixed at 30 days between November 2008 and December 2008 to meet the underlying assumption of population closure [23]. The closure test in program CAPTURE was used to test the assumption [24]–[26]. The sampling was carried out in two trapping sessions of 15 days each covering two blocks, each with 20 trap locations (Figure 1). The capture history data from the two blocks were combined and analyzed using program CAPTURE. Of the 40 trap locations, three became non-operational due to theft of cameras. Based on typical minimum home range sizes of leopards [27], [28], an average trap spacing of about 1.5 km was maintained to ensure that all individual leopards were potentially exposed to trapping. Each camera trap had two camera units facing each other 5 m apart, fixed at locations judged to be optimal leopard travel routes. Deercam DC 300 passive detection camera traps protected by metal shells were used and set at a height of 40–45 cm above the ground. Camera traps were checked twice daily to record the number of exposures, to change the films or batteries if needed, and to switch them on for night operation (around 6 pm to 7 am). The cameras were turned off during daytime because of the large amount of human and livestock traffic.

Individual leopards and hyaenas were identified from photos based on pelage marking patterns. However, in some cases we could not obtain photographs of both flanks of hyaenas, and therefore we used only the right flank to identify them.

We provide an index of trap visitation rates for other wild carnivores based on the maximum number of images obtained per species per trap site in all 37 traps during the survey period. In the absence of an appropriately designed survey for all the other species, and adequate samples of detections we could not use abundance models of occupancy [29]–[31].

### Estimating the Density of Leopards and Hyenas using Capture-recapture Sampling

Images of leopards more than a year old were used for estimating abundances [32] because the probability of capturing younger animals is very low (due to the time delay between first and second images if a family group pass a camera), and because the high mortality rate of this age class could lead to violation of population closure assumptions.

There are two different approaches to estimation of animal density from photographic capture-recapture data. The earlier approach, hereafter referred to as conventional capture recapture (CR) model [33], [34] relies on capture-frequency data derived from photographed animals to estimate population size (Appendix 1), and thereafter uses distances between locations of recaptures to obtain the effective area sampled for estimating density. However, recently this approach has been complimented by spatially explicit capture-recapture (SECR) models that incorporates information on capture location directly into the modeling and the estimation of capture probabilities [35], [36]. However, for the purpose of comparing our results with earlier studies (e.g. [27], [37], [38]), we present estimates based on both CR and SECR models. For estimating abundance with the CR models, we used the program CAPTURE [33], [39]. In CAPTURE, different models are compared based on expected effects of individual heterogeneity in capture probability (Model M_h_), behavioural responses to trapping (Model M_b_), and changes in capture probability among sampling occasions (Model M_t_) [34]. The null model assumes that capture probability is not affected by any of these factors. As suggested by Karanth & Nichols [40], we rejected the null model because the model assumption is likely to be violated due to territorial behaviour and trap response. We estimated the effective sampling area (for density estimates) by adding a circular buffer around each camera trap location equal to half the average mean maximum distance between locations of individuals photo-captured more than once [27], [37].

For more reliable estimation of leopard densities we preferred the recently developed spatially explicit capture-recapture models (SECR). In addition to individual animal capture histories, SECR models also use the spatial information from capture locations in the density estimation process (see [36] for a current summary of SECR models). Of the two types of SECR models available, we preferred the Bayesian models [35], [41] over the likelihood based models [42] because the former does not rest on asymptotic assumptions, and, can potentially be extended to open model scenarios to estimate survival and other parameters with multi-year data. For leopard density estimation we used program SPACECAP [36], which reports interval estimates of density as direct probabilities without asymptotic assumptions for our relatively small sample of captures [35]. Initially a buffer of 15 km was generated around a grid of equally spaced points (4757), each 0.336 km^2^ in size, to represent all probable leopard activity centers (see [35], [41] for a full description of the methods). An area of 1589 km^2^ leopard habitat over which these activity centers S_i_ were uniformly distributed was estimated after deducting an area of 2.67 km^2^ of water bodies. We used three input data files consisting of animal capture details (which individual was photographed at which site and on which sampling occasion), trap deployment details (which traps were active when and where) and the potential home range or activity centers. We used 52000 iterations, of which the initial 2000 were discarded, a thinning rate was set at 50, and augmentation of 110 individuals were used (see [35], [41] for details of estimation methodology).

Information on leopard deaths or captures that occurred at the study site, in the camera trapping period were obtained from the Forest Officials. Unlike most other parts of the world, government sanctioned lethal control is not the norm in India and leopards not killed legally.

## Results

### Leopard Density

A total of 4124 photo-exposures of 13 species were recorded during 1110 trap nights (Table 1). Humans were the most commonly photographed species, followed by domestic cats, leopards and striped hyenas (Table 1). A total of 81 leopard images were obtained of which five distinct adult males and six adult females could be identified. Two of the females were photographed with cubs and a third gave birth to one cub after we had radio-collared her six months later (Odden et al. unpublished data). Using program SPACECAP (SECR models) we obtained a posterior density estimate of 4.8 leopards per 100 km^2^ (sd = 1.2) (Table 2).

**Table 1:** The different species photographed over 30 days in November and December 2008 in the human-dominated landscape of Akole, the maximum number of each species in each trap pair summed over 37 traps, their status in the Schedules of the Indian Wildlife Protection Act and their IUCN status has been provided below.

Species	Total number of photo-capturesin 37 traps	Indian Wildlife Act Schedule	IUCN red list status
Leopard (*Panthera pardus* )	81	I	Near threatened
Rusty spotted cat (*Prionailurus rubiginosus*)	10	I	Vulnerable
Small Indian civet (*Viverricula indica*)	5	II	Least concern
Indian fox (*Vulpes bengalensis*)	1	II	Least concern
Jungle cat (*Felis chaus*)	20	II	Least concern
Striped hyaena (*Hyaena hyaena*)	65	III	Near threatened
Jackal (*Canis aureus indicus*)	3	III	Least concern
Black naped hare (*Lepus nigricollis*)	8	IV	Least concern
Human	830		
Domestic cat	147		
Domestic dog	12		
Mongoose (*Herpestes* spp)	1	IV	Least concern
Red wattled lapwing (*Vanellus indicus*)	1		Least concern

**Table 2:** The posterior summaries of the model parameters for n = 11 leopard individuals and n = 12 hyaena individuals.

Leopard	Posterior mean	Posterior SD	95% Lower HPD level	95% Upper HPD level
σ (5 Km)	0.3191	0.0892	0.1861	0.5044
Lam0	0.0522	0.0137	0.0249	0.076
B	0.035	4.0457	−8.0832	6.1183
Psi	0.6364	0.1625	0.3389	0.9478
N super	77.227	19.4256	43	117
Density	**4.8398**	**1.2174**	2.6948	7.3324
Hyaena				
σ (5 Km)	0.5756	0.2394	0.2194	1.0546
Lam0	0.026	0.0086	0.0117	0.0424
B	5.158	2.4752	0.6309	10.0645
Psi	0.6553	0.1749	0.3616	0.9834
N super	80.374	20.8174	46	120
Density	**5.037**	**1.3046**	2.8828	7.5204

The derived parameters are Lam0 which is the intercept of expected encounter frequency, σ is the “range parameter” of the species, B is the regression coefficient which measures the behavioural response, Psi is the ratio of the number of animals present within the space S to the maximum allowable number, Nsuper is the number of activity centres located in S, Density is Nsuper divided by S.

For the CR analyses (Table 3), we could not reject the null hypothesis of a closed population (z = 1.224, p = 0.889). The null model (Mo) was ranked first (Table 4) but the second best model (Mh) was regarded as the most appropriate due to ecological reasons described previously [34]. The average capture probability was relatively higher at 0.19 using the jackknife estimator, providing us with a population size of 12 leopards (SE = 1.46). All but one leopard individual were photographed more than once giving us a mean maximum distance estimate of 3.53 km moved between camera traps. Therefore, with an effectively sampled area of 187.5 km^2^, we obtained a leopard density of 6.4±0.78 (SE) per 100 km^2^ (Table 4). However, since spatially explicit CR are methods are clearly more reliable, our inferences are based on SECR results rather than conventional CR analyses, which are provided only for comparisons with earlier studies that use the older approach.

**Table 3:** Summary of photographic capture recapture sampling carried out in the human-dominated agricultural study site of Akole in December 2008.

	½ MMDM radius around individualtraps (leopard)	½ MMDM radius around individual traps (hyaena)
Total number of effective traps	37 pairs	37 pairs
Sampling occasions (number of days traps were set)	30 days	30 days
Trapping occasions	15	15
Sampling effort (number of days x sampling occasions)	1110	1110
Estimated buffer width (1/2 MMDM around each trap)	1.76 km	1.85 km
Number of captures and recaptures n	34	22
Number of individuals captured (Mt+1)	11	12
Estimated number of leopards using model Mh and using thejack knife estimator	12±1.46	18±6.48
Estimated number of leopards using 95% CI	12–19	14–45
Minimum convex area around camera traps	136 km^2^	136 km^2^
Effective Area sampled	187.54 km^2^	193.44 km^2^
Estimated leopard density (**±** SE) in 100 km^2^	**6.4±0.78**	**9±3.35**

MMDM = Mean Maximum Distance Moved, i.e. the average maximum distance between locations of recaptured individuals.

**Table 4:** Capture probabilities for leopards and hyaenas based on different models.

Model	M(o)	M(h)	M(b)	M(bh)	M(t)	M(th)	M(tb)	M(tbh)
**leopard**	1	0.87	0.42	0.69	0	0.43	0.4	0.71
**hyaena**	1	0.85	0.43	0.71	0	0.46	0.38	0.71

The different models are Mo (null model where every individual has the same capture probability), Mt (capture probability varies with the sampling occasion), Mb (capture probability differs between individuals who have been photographed before and those that have been not), the others are combinations of the above. The CAPTURE program uses a discriminant function to provide the best model based on a discriminant function.

No leopards were found dead or captured in the camera trapping period. However, between November 2005 and March 2009, ten leopards were found dead in the study area (2 cubs, six females and 2 males), two more adults (sex unknown) had fallen in wells but had escaped during the rescue process, and an adult male and female were captured and translocated long distance (more than 200 km away).

### Hyaena Density

In the case of hyaenas, we obtained 65 images of which 26 were usable. A total of 12 individuals could be recognized from the stripe patterns. Using the SECR analysis (program SPACECAP), we obtained a density estimate of 5.03 hyaenas per 100 km^2^ (sd = 1.3). In the CR analyses (program CAPTURE) the null hypothesis of a closed population for the hyaenas could not be rejected (z = 1.237, p = 0.89). Again, in the case of hyaenas we used the Mh model although it was ranked as second best, following the M_0_ model. The average capture probability per camera trap per night was 0.098, and thus lower than for leopards, and we obtained a population size estimate of 15 with a wider SE of 3.6.

Using the ½ MMDM buffer around each camera trap location for estimating effective sampling area, the hyaena density estimate was 9±3.35 (SE)/100 km^2^.

## Discussion

Photographic capture recapture has been used for estimating densities of many secretive large carnivore species, including tigers [34], [43], [44], leopards [27], [38], [44], snow leopards (*Panthera uncia)* [45], jaguars (*Panthera onca)* [46] and hyaenas [47]–[49]. The previous conventional analyses of obtaining population density have given way to more robust methods that use the spatial information of location of traps rather than methods that estimate the size of the effective area heurestically [35], [41]. The spatial capture recapture model is also less affected by the small sample sizes often associated with camera trap data from large felids [35], [41].

A range of studies have estimated leopard and hyaena densities using photographic capture-recapture. Balme *et al.* [27] obtained a density of 7.17 per 100 km^−2^ in a protected buffer area in South Africa while the non-protected farmlands had leopards at 2.49/100 km^−2^. A recent study from a protected area in Cambodia, devoid of human habitations, obtained leopard density estimates of 3.6/100 km^2^ and 3.8/100 km^2^ using SECR (Spatially Explicit Capture Recapture) and conventional CR (Capture Recapture) methods [50]. Estimate of leopard density in India is available from only within protected areas with 15/100 km^2^ [38] but this area is largely devoid of people and agricultural land-use. Harihar *et al.* [44] found that the density of leopards in the Rajaji National Park decreased from 9.76/100 km^2^ to 2.07/100 km^2^ with a concurrent increase of tiger populations following relocation of people from within the Park. Recent estimates from camera trapping studies on hyaenas outside protected areas in India report density of 3.67–6.5/100 km^2^ [48] whereas higher densities of 15.1/100 km^2^ [47] and 3.9–5.67/100 km^2^ [49] were obtained from within the protected areas of Sariska Tiger Reserve and Rajaji National Park, India.

Prey biomass is seen to strongly influence tiger [51] and leopard [10], [52] density although other factors such as interspecific competition [44] and disease [53] also affect carnivore densities. Leopards in protected areas in India feed on small to medium sized wild prey such as cheetal (*Axis axis)*, sambar (*Rusa unicolor)* and langur (*Semnopithecus* spp.) [54], [55]. Our study site contains no other apex predator and no wild ungulate prey species suitable for leopards. Data (unpublished results) indicates that leopards in our study area primarily subsist on a diet of domestic dogs and livestock, which are abundant. Thus in our case, models of predator-prey density need to include estimates of the density of domestic prey species as well as wild prey.

We also obtained clear evidence in the camera-trap photos, and from cubs that were found dead or rescued from wells in the study area, that the study population consisted of resident and reproductive individuals and was not made up of only sub-adult animals which had potentially dispersed from some distant patch of forest habitat. These lines of evidence along with information from collared leopards (unpublished results) combine to overturn the popular view that leopards in such human-dominated habitats represent a few occasional “stray” individuals. It is clear that the study area contained a dense, established, breeding population of leopards.

The results are also interesting for evolving theories on the ways in which community structure changes following human interventions [4], [56]. Conventional theory would not have predicted the persistence of such a carnivore biased community with eight species of carnivore persisting in a high density human use landscape devoid of wild herbivore species. The leopard is the apex predator here with the tiger and the wolf being absent from the system, although wolves have been reported in the dry hills surrounding the irrigated valley where this study was conducted. This merely underlines how aspects of carnivore behavior that permit adaptation to diverse habitats are often just as crucial as the more conventional life-history metrics such as body size and reproductive rates that are often used to predict species and community persistence [4], [56].

Our results show that large predators, like leopards and striped hyaena, are probably not very suitable as either flagships, umbrellas or indicator species for wild nature in India. It appears that sugarcane and other tall crops, domestic dogs and livestock are sufficient as habitat and prey, respectively, for the leopards outside designated protected areas. In these contexts, leopards can serve as important flagships under an alternative approach in the conservationist’s toolkit. This is the philosophy based on sharing space and integrating wildlife into human-modified landscapes where the focus is as much on knowing the “social carrying capacity”, which is defined by the tolerance of humans towards predators [57], in addition to the ecological carrying capacity. This will require an acceptance of situations where humans and wildlife share multi-use landscapes to the extent possible, outside of protected areas. To achieve this, the research focus must include areas outside protected areas as much as those inside, and social science research as much as ecological research. It also requires enabling flexible and pragmatic legislation acceptable to rural people because it needs to take into account their concerns and interests also. This study shows that leopards can persist in the human-modified landscapes and is possibly dependent mainly on the social tolerance. Despite their subsistence on a diet of domestic animals the levels of conflict in the study area are quite low (unpublished results). Although leopards are implicated in the highest number of fatal attacks by a large felid on humans in some other parts of India, no fatal attacks are known from the study area, even though more than 300 people/km^2^ share the same space with 5–6 leopards/100 km^2^. There are indications from other parts of India that this phenomena of carnivores surviving in highly modified landscapes is not confined to leopards. The Asiatic lion (*Panthera leo*) has recently extended its range and is known to now use even the areas outside the protected area [17] as well as some tiger populations which have recently been reported to use sugarcane areas [58] in northern India.

Our study documents for the first time that a whole guild of predators can persist in totally human dominated landscape in India. This probably has a lot to do with India's laws which makes it illegal to kill any wildlife for sport or for consumption. In the case of large cats, even killing man-eaters requires permission from the state authorities, unlike most other countries where even livestock killers are often removed immediately. India has also been known for its tolerance towards other life forms, even the large, potentially dangerous species. It also implies that a much greater area of potential "tolerance habitat" is available outside protected areas, and potentially a far greater degree of connectivity between protected areas than generally expected. The ability of conflict causing species to persist in close proximity to humans greatly expands the spatial extent of human – wildlife interfaces beyond the narrow “zone of influence” [20] that surrounds protected areas. This poses many challenges for India’s legislation and wildlife management structures which are heavily focused on protected areas and are very wildlife-centric. There is a clear need to recognize that these potentially conflict causing species [59] can, and will, colonise many areas and that their management cannot only be based on a hands-off policy. That being said there is a clear need to ensure that management interventions do not make the situations any worse [18].

The results of this study add to an emerging body of empirical results that demonstrate the conservation value of unprotected, human-dominated landscapes for large carnivores [60]–[64] as an important supplement to protected areas. While this approach has long been recognized in temperate areas (e.g. [65], [66]), it is only recently being demonstrated in tropical areas [67], although its general applicability as a conservation model may be highly species and context specific [62] depending on a range of ecological, social, cultural and economic factors.

## Supporting Information

Appendix S1 **Capture history matrices for leopard and hyaena.**
(DOC)

## References

[1] Kruuk H (2003) Hunter and Hunted. Cambridge, U.K. : Cambridge University Press, 264 p.

[2] Caro T, O'Doherty G (1999) On the use of surrogate species in conservation biology. Conservation Biology 13: 805–814.

[3] Linnell JDC, Swenson JE, Andersen R (2000) Conservation of biodiversity in Scandinavian boreal forests: Large carnivores as flagships, umbrellas, indicators, or keystones? Biodiversity and Conservation 9: 857–868.

[4] Dalerum F, Somers MJ, Kunkel K, Cameron EZ (2008) The potential for large carnivores to act as biodiversity surrogates in southern Africa. Biodiversity and Conservation 17: 2939–2949.

[5] Terborgh J (1999) Requiem for Nature. Washington, D.C. : Island Press. 234 p.

[6] Woodroffe RB (2000) Predators and people: using human densities to interpret declines of large carnivores. Animal Conservation 3: 165–73.

[7] Karanth KU, Gopal R (2005) An ecology-based policy framework for human-tiger coexistence in India. In: Woodroffe R, Thirgood S, Rabinowitz A, editors. People and wildlife: Conflict or coexistence? Cambridge: Cambridge University Press. 373–387.

[8] Walston J, Robinson JG, Bennett EL, Breitenmoser U, da Fonseca GAB, et al (2010) Bringing the Tiger Back from the Brink–The Six Percent Solution. PLoS Biology 8(9): e1000485 doi:10.1371/journal.pbio.1000485 20856904 10.1371/journal.pbio.1000485 PMC2939024

[9] Carbone C, Pettorelli N. Stephens PA (2010) The bigger they come, the harder they fall: body size and prey abundance influence predator–prey ratios. Biology Letters 7: 312–315. 21106569 10.1098/rsbl.2010.0996 PMC3061189

[10] Carbone C, Gittleman JL (2002) A common rule for the scaling of carnivore density. Science 295: 2273–2276. 11910114 10.1126/science.1067994

[11] Hayward MW, O’Brien J, Kerley GIH (2007) Carrying capacity of large African predators: predictions and tests. Biological Conservation 139: 219–229.

[12] Maddox T (2003) The ecology of cheetahs and other large carnivores in a pastoralist-dominated buffer zone. Ph.D. University College & Institute of Zoology, London, U.K.

[13] Sanderson EW, Redford KH, Vedder A, Ward SE, Coppolillo PB (2002) A conceptual model for conservation planning based on landscape species requirements. Landscape and Urban Planning 58: 41–56.

[14] Mech LD, Boitani L (2003) Wolves. Behaviour Ecology and Conservation. Chicago: The University of Chicago Press. 472 p.

[15] Gehrt SD, Riley SPD, Cypher BL, editors (2010) Urban Carnivores: Ecology, Conflict, and Conservation. Baltimore: The Johns Hopkins University Press. 285 p.

[16] Jhala YV, Giles RH (1991) The status and conservation of the wolf in Gujarat and Rajasthan, India. Conservation Biology 5: 476–483.

[17] Banerjee K, Jhala YV, Pathak B (2010) Demographic structure and abundance of Asiatic lions Panthera leo persica in Girnar Wildlife Sanctuary, Gujarat, India. Oryx 44: 248–251.

[18] Athreya V, Odden M, Linnell JDC, Karanth KU (2011) Translocation as a Tool for Mitigating Conflict with Leopards in Human-Dominated Landscapes of India. Conservation Biology 25: 133–141. 21054526 10.1111/j.1523-1739.2010.01599.x

[19] Athreya V, Thakur SS, Chaudhuri S, Belsare AV (2007) Leopards in human–dominated areas: a spillover from sustained translocations into nearby forests? Journal of Bombay Natural History Society 104: 45–50.

[20] DeFries R, Karanth KK, Pareeth S (2010) Interactions between protected areas and their surroundings in human-dominated tropical landscapes. Biological Conservation 143: 2870–2880.

[21] Working Plan (2010) Working Plan for Ahmednagar Forest Division and Sangamner Forest sub-division of Nashik Circle. Maharashtra Forest Department. 298 p.

[22] Karanth KU, Nichols JD, Kumar NS, Jathanna D (2011) Estimating demographic parameters in a tiger population from long term camera-trap data. In: O’Connel AF, Nichols JD, Karanth KU, editors. Camera Traps in Animal Ecology. Tokyo: Springer. 145–162.

[23] Williams BK, Nichols JD, Conroy MJ (2002) Analysis and management of animal populations. San Diego: Academic Press. 818 p.

[24] Stanley TR, Burnham KP (1999) A closure test for time-specific capture–recapture data. Environmental and Ecological Statistics 6: 197–209.

[25] White GC, Anderson DR, Burnham KP, Otis DL (1982) Capture–recapture removal methods for sampling closed populations. New Mexico: Los Alamos National Laboratory Publication. 235 p.

[26] Rexstad E, Burnham KP (1992) User’s guide for interactive program CAPTURE: Abundance estimation of closed animal populations. Available http://www.mbr-pwrc.usgs.gov/software/doc/capturemanual.pdf. Accessed 25 January 2011.

[27] Balme GA, Hunter LTB, Slotow R (2009) Evaluating Methods for Counting Cryptic Carnivores. Journal of Wildlife Management 73: 433–441.

[28] Odden M, Wegge P (2005) Spacing and activity patterns of leopards Panthera pardus in the Royal Bardia National Park, Nepal. Wildlife Biology 11: 145–152.

[29] Sollmann R, Furtado MM, Gardner B, Hofer H, Jácomo AT, et al (2011) Improving density estimates for elusive carnivores: accounting for sex-specific detection and movements using spatial capture–recapture models for jaguars in central Brazil. Biological Conservation 144: 1017–1024.

[30] Royle JA, Nichols JD (2003) Estimating abundance from repeated presence-absence data or point counts. Ecology 84: 777–790.

[31] Gopalaswamy AM, Karanth KU, Kumar NS, Macdonald DW (2012b) Estimating tropical forest ungulate densities from sign surveys using abundance models of occupancy. Animal Conservation 15: 669–679.

[32] Karanth KU, Nichols JD (2010) Non-invasive survey methods for assessing tiger populations. In: Tilson RL, Nyhus PJ, editors. Tigers of the world: Science, politics and conservation of Panthera tigris. New York: Elsevier. 241–261.

[33] Otis DL, Burnham KP, White GC, Anderson DR (1978) Statistical inference from capture data on closed animal populations. Wildlife Monographs 62: 1–135.

[34] Karanth KU, Nichols JD (1998) Estimation of tiger densities in India using photographic captures and recaptures. Ecology 79: 2852–2862.

[35] Royle JA, Karanth KU, Gopalaswamy AM, Kumar NS (2009a) Bayesian inference in camera trapping studies for a class of spatial capture-recapture models. Ecology 90: 3233–3244. 19967878 10.1890/08-1481.1

[36] Gopalaswamy AM, Royle AJ, Hines JE, Singh P, Jathanna D, et al (2012a) Program SPACECAP: software for estimating animal density using spatially explicit capture–recapture models. Methods in Ecology and Evolution 3: 1067–1072.

[37] Henschel P, Ray J (2003) Leopards in African rainforests: Survey and monitoring techniques. Wildlife Conservation Society. (http://www.savingwildplaces.com/swp-globalcarnivore). 50 p.

[38] Harihar A, Pandav B, Goyal SP (2009) Density of leopards (Panthera pardus) in the Chilla Range of Rajaji National Park, Uttarakhand, India. Mammalia 73: 68–71.

[39] Burnham K, Rexstad E (1993) Modeling heterogeneity in Survival Rates of Banded Waterfowl. Biometrics 49: 1194–1208.

[40] Nichols JD, Karanth KU (2002) Nichols: Statistical concepts: estimating absolute densities of tigers using capture-recapture sampling. In: Karanth KU, Nichols JD, editors. Monitoring tigers and their prey: a manual for wildlife researchers, managers and conservationists in tropical Asia. Center for Wildlife Studies, Bangalore, India, 139–152.

[41] Royle JA, Nichols JD, Karanth KU (2009b) A hierarchical model for estimating density in camera-trap studies. Journal of Applied Ecology 46: 118–127.

[42] Borchers DL, Efford MG (2008) Spatially explicit maximum likelihood methods for capture–recapture studies. Biometrics 64: 377–385. 17970815 10.1111/j.1541-0420.2007.00927.x

[43] Karanth KU, Nichols JD, Kumar NS, Hines JE (2006) Assessing tiger population dynamics using photographic capture–recapture sampling. Ecology 87: 2925–2937. 17168036 10.1890/0012-9658(2006)87[2925:atpdup]2.0.co;2

[44] Harihar A, Pandav B, Goyal SP (2011) Responses of leopard Panthera pardus to the recovery of a tiger Panthera tigris population. Journal of Applied Ecology 48: 806–814.

[45] Janečka JE, Munkhtsog B, Jackson RM, Naranbaatar G, Mallon DP, et al (2011) Comparison of noninvasive genetic and camera-trapping techniques for surveying snow leopards. Journal of Mammalogy 92: 771–783.

[46] Soisalo MK, Cavalcanti SMC (2006) Estimating the density of a jaguar population in the Brazilian Pantanal using camera-traps and capture–recapture sampling in combination with GPS radio-telemetry. Biological Conservation 129: 487–496.

[47] Gupta S, Mondal K, Sankar K, Qureshi Q (2009) Estimation of striped hyaena (Hyaena hyaena) population using camera traps in Sariska Tiger Reserve, Rajasthan India. Journal of Bombay Natural History Society 106: 284–288.

[48] Singh P, Gopalaswamy AM, Karanth KU (2010) Factors influencing densities of striped hyenas (Hyaena hyaena) in arid regions of India. Journal of Mammalogy 91: 1152–1159.

[49] Harihar A, Ghosh M, Fernandes M, Pandav B, Goyal SP (2010) Use of photographic capture-recapture sampling to estimate density of Striped Hyena (Hyaena hyaena): implications for conservation. Mammalia 74: 83–87.

[50] Gray TNE, Prum S (2011) Leopard density in post-conflict landscape, Cambodia: Evidence from spatially explicit capture-recapture. The Journal of Wildlife Management 76: 163–169.

[51] Karanth KU, Nichols JD, Kumar NS, Link WA, Hines JE (2004) Tigers and their prey: predicting carnivore densities from prey abundance. PNAS 14: 4854–4858. 10.1073/pnas.0306210101 PMC387338 15041746

[52] Khorozyan IG, Malkhasyan AG, Abramov AV (2008) Presence-absence surveys of prey and their use in predicting leopard (Panthera pardus) densities: a case study from Armenia. Integrative Zoology 3: 322–332. 21396082 10.1111/j.1749-4877.2008.00111.x

[53] Chauvenet ALM, Durant SM, Hilborn R, Pettorelli N (2011) Unintended Consequences of Conservation Actions: Managing Disease in Complex Ecosystems. PLoS ONE 6(12): e28671 doi:10.1371/journal.pone.0028671 22163323 10.1371/journal.pone.0028671 PMC3233597

[54] Karanth KU, Sunquist ME (1995) Prey selection by tiger, leopard and dhole in tropical forests. Journal of Animal Ecology 64: 439–450.

[55] Ramesh T, Snehalatha V, Sankar K, Qureshi Q (2009) Food habits and prey selection of tiger and leopard in Mudumalai Tiger Reserve, Tamil Nadu, India. Journal of Scientific Transactions in Environment and Technovation 2: 170–181.

[56] Woodroffe RB, Ginsberg JR (2005) King of the Beasts? Evidence for Guild Redundancy among Large Mammalian Carnivores. In: Ray JC, Berger J, Redford KH, Steneck R, editors. Large Carnivores and Biodiversity: Does saving one conserve the other? Washington DC: Island Press. 154–178.

[57] Breitenmoser U, Angst C, Landry JM, Breitenmoser-Wursten C, Linnell JDC, et al.. (2005) Non-lethal techniques for reducing depredation. In Woodroffe R, Thirgood S, Rabinowitz A, eds. People and Wildlife. Conflict or Co-existence? New York: Cambridge University Press. 49–71.

[58] Wikramanayake E, McKnight M, Dinerstein E, Joshi A, Gurung B, et al (2004) Designing a conservation landscape for tigers in human-dominated environments. Conservation Biology 18: 839–844.

[59] Loveridge AJ, Hemson G, Davidson Z, MacDonald DW (2010) African Lions on the edge: reserve boundaries as ‘attractive sinks’. In: MacDonald DW, Loveridge AJ, editors. Biology and Conservation of Wild Felids. Oxford: Oxford University Press. 283–304.

[60] Maehr DS (1990) The Florida panther and private lands. Conservation Biology 4: 167–170.

[61] Negroes N, Revilla E, Fonseca C, Soares AMVM, Jacomo ATA, et al (2011) Private forest reserves can aid in preserving the community of medium and large-sized vertebrates in the Amazon arc of deforestation. Biodiversity Conservation. 20: 505–518.

[62] Daily GC, Ceballos G, Pacheco J, Suzan G, Sanchez-Azofeifa A (2003) Countryside biogeography of neotropical mammals: conservation opportunities in agricultural landscapes of Costa Rica. Conservation Biology. 17: 1814–1826.

[63] Rosas-Rosas OC, Valdez R (2010) The Role of Landowners in Jaguar Conservation in Sonora, Mexico. Conservation Biology 24: 366–371. 20136872 10.1111/j.1523-1739.2009.01441.x

[64] Mazzolli M (2010) Mosaics of Exotic Forest Plantations and Native Forests as Habitat of Pumas. Environmental Management 46: 237–253. 20665214 10.1007/s00267-010-9528-9

[65] Linnell JDC, Swenson JE, Andersen R (2001a) Predators and people: conservation of large carnivores is possible at high human densities if management policy is favourable. Animal Conservation 4: 345–350.

[66] Linnell JDC, Andersen R, Kvam T, Andrén H, Liberg O, et al (2001b) Home range size and choice of management strategy for lynx in Scandinavia. Environmental Management 27: 869–879. 11393321 10.1007/s002670010195

[67] Stein AB, Fuller TK, DeStefano S, Marker LL (2011) Leopard population and home range estimates in north-central Namibia. African Journal of Ecology 49: 383–387.

