# Supplementary material for: Big Cats in Our Backyards: Persistence of Large Carnivores in a Human Dominated Landscape in India
Source: PLoS One. 2013 Mar 6;8(3):e57872. doi: 10.1371/journal.pone.0057872 (PMC3590292; doi:10.1371/journal.pone.0057872)
Supplement: Appendix S1 — Capture history matrices for leopard and hyaena. (DOC) [file pone.0057872.s001.doc]

**Appendix 1**. Capture history matrices for leopard and hyaena. The ID consists of different individuals identified from their pelage patterns over 15 sampling occasions, spread over 2 blocks in 30 days in December 2008. The 0 represents no animal photographed and 1 denotes the presence of the individual on the given sampling occasion.

| **Leopard ID** | | **Sampling Occasions** | | | | | | | | | | | | | | | | | | | | | |
| --- | --- | --- | --- | --- | --- | --- | --- | --- | --- | --- | --- | --- | --- | --- | --- | --- | --- | --- | --- | --- | --- | --- | --- |
|  |  | **1** | | **2** | | **3** | | **4** | | **5** | | **6** | | **7** | | **8** | **9** | **10** | **11** | **12** | **13** | **14** | **15** |
| **AkA** | | 1 | | 0 | | 1 | | 0 | | 0 | | 0 | | 1 | | 0 | 0 | 0 | 0 | 0 | 0 | 0 | 0 |
| **AkB** | | 1 | | 0 | | 0 | | 0 | | 0 | | 0 | | 0 | | 0 | 0 | 0 | 0 | 0 | 1 | 0 | 0 |
| **AkC** | | 0 | | 1 | | 0 | | 0 | | 1 | | 1 | | 0 | | 0 | 0 | 0 | 0 | 0 | 0 | 0 | 1 |
| **AkD** | | 0 | | 1 | | 0 | | 1 | | 0 | | 0 | | 1 | | 0 | 0 | 0 | 0 | 0 | 1 | 0 | 0 |
| **AkE** | | 0 | | 0 | | 0 | | 1 | | 0 | | 1 | | 0 | | 0 | 0 | 0 | 0 | 0 | 0 | 0 | 1 |
| **AkF** | | 0 | | 0 | | 0 | | 0 | | 1 | | 1 | | 0 | | 1 | 0 | 0 | 0 | 1 | 0 | 0 | 0 |
| **AkG** | | 0 | | 0 | | 0 | | 1 | | 0 | | 0 | | 0 | | 0 | 0 | 0 | 1 | 0 | 0 | 0 | 0 |
| **AkH** | | 1 | | 0 | | 1 | | 0 | | 0 | | 0 | | 0 | | 0 | 0 | 0 | 0 | 0 | 1 | 0 | 1 |
| **AkI** | | 0 | | 0 | | 1 | | 1 | | 0 | | 0 | | 0 | | 0 | 0 | 0 | 1 | 0 | 1 | 0 | 1 |
| **AkJ** | | 0 | | 0 | | 0 | | 0 | | 1 | | 1 | | 0 | | 0 | 0 | 0 | 0 | 0 | 0 | 0 | 0 |
| **AkK** | | 0 | | 0 | | 0 | | 0 | | 0 | | 0 | | 0 | | 0 | 0 | 0 | 0 | 0 | 0 | 1 | 0 |
| **Hyaena ID** | **Sampling Occasions** | | | | | | | | | | | | | | | | | | | | | | |
|  | **1** | | **2** | | **3** | | **4** | | **5** | | **6** | | **7** | | **8** | | **9** | **10** | **11** | **12** | **13** | **14** | **15** |
| **AkA** | 1 | | 1 | | 0 | | 0 | | 0 | | 1 | | 0 | | 0 | | 0 | 0 | 0 | 0 | 1 | 0 | 0 |
| **AkB** | 0 | | 0 | | 1 | | 0 | | 0 | | 0 | | 0 | | 0 | | 0 | 0 | 0 | 0 | 0 | 0 | 0 |
| **AkC** | 0 | | 0 | | 0 | | 0 | | 1 | | 0 | | 0 | | 1 | | 0 | 0 | 0 | 0 | 1 | 0 | 0 |
| **AkD** | 0 | | 1 | | 0 | | 0 | | 1 | | 0 | | 0 | | 0 | | 0 | 0 | 0 | 0 | 1 | 0 | 0 |
| **AkE** | 0 | | 0 | | 0 | | 0 | | 0 | | 0 | | 1 | | 0 | | 0 | 0 | 0 | 0 | 0 | 0 | 0 |
| **AkF** | 0 | | 0 | | 0 | | 0 | | 0 | | 0 | | 0 | | 0 | | 1 | 0 | 0 | 0 | 0 | 0 | 1 |
| **AkG** | 0 | | 1 | | 0 | | 0 | | 0 | | 0 | | 0 | | 0 | | 0 | 0 | 0 | 0 | 0 | 0 | 0 |
| **AkH** | 0 | | 0 | | 0 | | 0 | | 0 | | 0 | | 0 | | 1 | | 0 | 0 | 0 | 0 | 0 | 0 | 0 |
| **AkI** | 0 | | 0 | | 0 | | 0 | | 0 | | 0 | | 0 | | 0 | | 0 | 1 | 0 | 0 | 0 | 1 | 0 |
| **AkJ** | 0 | | 0 | | 0 | | 0 | | 0 | | 0 | | 0 | | 0 | | 0 | 0 | 1 | 0 | 0 | 0 | 0 |
| **AkK** | 0 | | 0 | | 0 | | 0 | | 0 | | 0 | | 0 | | 0 | | 0 | 1 | 0 | 0 | 0 | 0 | 0 |
| **AkL** | 0 | | 1 | | 0 | | 0 | | 0 | | 0 | | 0 | | 0 | | 0 | 0 | 1 | 0 | 0 | 0 | 0 |
